# Investigating the Microscopic Mechanism of Ultrasonic-Vibration-Assisted-Pressing of WC-Co Powder by Simulation

**DOI:** 10.3390/ma16145199

**Published:** 2023-07-24

**Authors:** Yuhang Chen, Yun Wang, Lirong Huang, Binbin Su, Youwen Yang

**Affiliations:** 1 School of Mechanical and Electrical Engineering, Jiangxi University of Science and Technology, Ganzhou 341000, China; 2 Jiangxi Province Key Laboratory of Maglev Technology, School of Electrical Engineering and Automation, Jiangxi University of Science and Technology, Ganzhou 341000, China

**Keywords:** WC-Co powder, ultrasonic-vibration-assisted pressing process, finite element simulation, pressed billet density

## Abstract

The ultrasonic-vibration-assisted pressing process can improve the fluidity and the uneven distribution of density and particle size of WC-Co powder. However, the microscopic mechanism of ultrasonic vibration on the powder remains unclear. In this paper, WC particles with diameter 5 μm and Co particles with diameter 1.2 μm were simulated by three-dimensional spherical models with the aid of the Python secondary development. At the same time, the forming process of the powder at the mesoscale is simulated by virtue of the finite element analysis software ABAQUS. In the simulation process, the vibration amplitude was set to 1, 2, and 3 μm. Their influence on the fluidity, the filling density, and the stress distribution of WC-Co powder when the ultrasonic vibration was applied to the conventional pressing process was investigated. The simulation results show that the ultrasonic vibration amplitude has a great influence on the density of the compact. With an increase in the ultrasonic amplitude, the compact density also increases gradually, and the residual stress in the billet decreases after the compaction. From the experimental results, the size distribution of the billet is more uniform, the elastic after-effect is reduced, the dimensional instability is improved, and the density curves obtained by experimentation and simulation are within a reasonable error range.

## 1. Introduction

WC-Co-cemented carbide with high hardness, high toughness, high wear resistance, and other excellent properties is widely used in many fields, such as mining, aerospace, automobile manufacturing, oil drilling, and so on [1,2]. With the continuous improvement in social production levels, the demand for cemented carbide in the manufacturing industry is increasing. However, in the actual production process, various defects often appear in the cemented carbide billet, such as delamination, fractures, missing angles, etc. because of the poor powder fluidity and the uneven distribution of density and particle size, which greatly limits the application of its products in various fields [3]. Improving the uniformity of the billet density and particle size distribution of cemented carbide can not only improve the hardness, bending strength, fracture toughness and other comprehensive mechanical properties, but also improve the physical properties of the material itself, such as electrical conductivity, thermal conductivity, permeability, and thermal expansion coefficient, and high-density powder metallurgy materials can also result in parts having a better machining performance and machining surface [4,5,6].

As a result, in order to improve the process of powder metallurgy, reduce the rejection rate, and improve the comprehensive performance of products, domestic and foreign researchers have carried out much research on the movement law of powder. The research shows that the particle will produce a volume effect, surface effect, and particle convection phenomenon under the ultrasonic-vibration-assisted pressing process [7,8]. Moreover, the ultrasonic-vibration-assisted pressing process can improve the fluidity and the uneven distribution of density and particle size of WC-Co powder, which is beneficial in modeling the arrangement of particles/bulk solids. Alhazaa used vibrations in diffusion bonding and sintering during impulse-pressure-assisted diffusion bonding and found that varying pressure could indeed reduce bonding times in diffusion bonding and reduce the requirements for pre-bonding surface preparation [9]. Sliva found that containers had different cross-section shapes, but their cross-section area and weight were constant because of the optimum arrangement of spherical particles [10]. Zhao Yanbo et al. [11] studied the influence of vibration frequency and vibration amplitude on the filling effect of iron powder and obtained the best parameters for the filling effect. Zuo Miaomiao et al. [8] analyzed the differences between the internal contact force of granular medium and the force chain, porosity, and coordination number in the particle medium after the action of the ultrasonic vibration. Wang Wentao et al. [12] studied the effect of vibration on the dense packing of refractory powder and determined that the average porosity of powder first decreased and then increased with the increase in vibration time, and they pointed out that the compaction effect caused by longitudinal vibration was better than that caused by transverse vibration. Sedaghat et al. [13] proposed a physics-based constitutive model to accurately describe the deformation behavior in the process of ultrasonic-vibration-assisted forming. At the same time, the finite element method was used to conduct a numerical simulation of the upsetting and progressive forming to evaluate the accuracy of the model. From the results, by considering the dislocation dynamics and acoustic energy transfer mechanism in the material under the ultrasonic vibration, the newly established ultrasonic constitutive equation could accurately predict the acoustic–plastic behavior of the material. Meanwhile, the application of ultrasonic vibration could significantly reduce the flow stress of the material, making it become soft during the forming process. The larger the amplitude, the smaller the inflow stress. Liu Bo et al. [14] used discrete element software to simulate the influence of Nd/Fe/B permanent magnet powder on the filling density at different vibration frequencies and vibration times. The results showed that the filling density reached its maximum when the vibration frequency was between 66 and 70 Hz, and the density first increased and then stabilized with the increase in vibration time. However, there have been few studies on the pressing process of WC-Co-cemented carbide by the ultrasonic-vibration-assisted pressing process.

This work proposes the use of the ABAQUS finite element software and Python co-simulation to simulate the pressing process of WC-Co powder with the aid of ultrasonic vibration. Compared with conventional pressing, the effects of changes in the vibration amplitude on the evolution of the fluidity, filling density, and stress distribution of WC-Co powder after application of the ultrasonic vibration are explored, and the result of the pressing experiment is compared with that of the simulation.

## 2. Construction of the Experiment Platform

**Figure 1:**
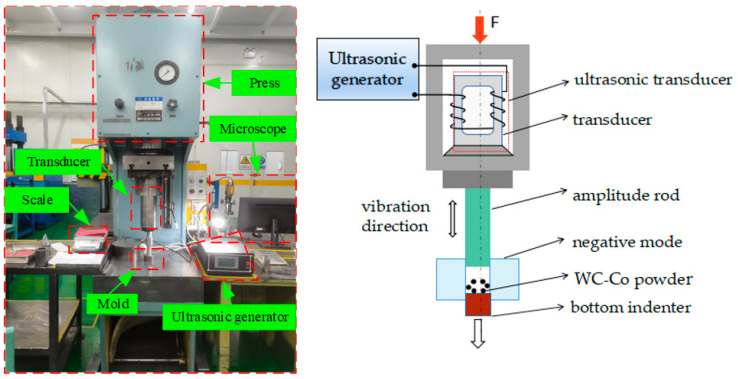
The experiment platform of the ultrasonic-vibration-assisted pressing process and its schematic drawing.

An experiment platform was constructed to apply the axial ultrasonic vibration to the traditional pressing process, as shown in Figure 1. This platform is primarily made up of five parts: an automatic press, an ultrasonic transducer, an ultrasonic generator, a mold set, and a piece of rubber cushion. The ultrasonic transducer is composed of a piezoelectric ceramic transducer, which can convert electrical energy into mechanical energy, an amplitude rod, and a tool head. The vibration frequency is 20 kHz and the driving power is 2 kW, and the ultrasonic amplitude can be steplessly adjusted from 0% to 100%. It is mounted on the automatic press with a flange whose pressure is transmitted directly to the transducer shell.

First, a certain mass of mixture of WC-Co powder is weighed and poured into the mold. When the mold is filled with powder, the automatic press is started to make its indenter move slowly until it comes into contact with the powder. Then, the ultrasonic transducer is started, and certain pressure is applied for a few seconds for the prepressure ultrasound. Finally, the pressure is applied slowly until the working pressure is attained. In order to calculate the density, the mass, the height and the diameter of the pressed billet are measured, and the errors of the mass and size are ±1 mg and ±0.01 mm, respectively.

## 3. Construction of the Mathematical Model

The continuum theory and the discrete element method are commonly used in the numerical simulation of powder materials [15]. The continuum theory has contributed to many achievements in the research on powder impact forming, which mainly regards powder as a continuum to study the dynamic mechanical response of powder in the loading process [16]. Compared with the continuum theory, the discrete element method regards powder particles as independent discrete individuals, and each discrete individual has corresponding physical properties, so the discrete element method is more in line with the actual situation. In this paper, the numerical simulation of WC-Co powder pressing process is carried out based on the discrete element method, according to Newton’s second law, so the equation of motion can be obtained as shown in Equation (1):

(1) $$M\frac{d^{2}X}{dt^{2}}+C\frac{dX}{dt}+KX(t)=f(t)$$

*M*—the mass of the particle, kg; *X*—the displacement of the particle, m; *C*—the damping coefficient; *K*—the elastic coefficient; *t*—time, s; *f*—the unit load, N.

**Figure 2:**
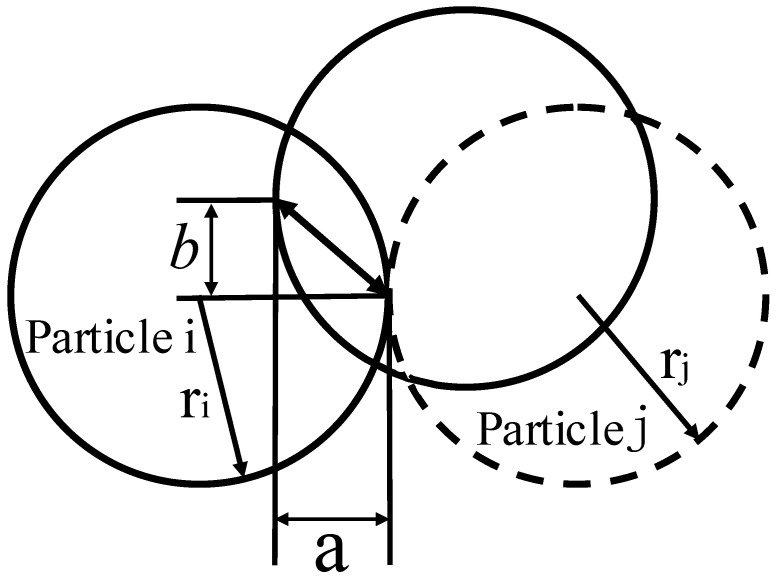
The contact slip between particles.

The role of the ultrasonic-vibration-assisted powder pressing process is to make the powder particles inside the mold undergo violent collision contact through high-frequency vibration, resulting in greater mobility, so that some smaller particles are evenly filled into the pores, with the aim of improving the density of the billet and reducing its porosity [8]. In order to better fit the actual situation and reduce the calculation amount, the soft-ball model is used to calculate the contact force between particles. The model structure is shown in Figure 2. Particle i and particle j have contact slip under the external forces. The dotted line represents the particles just in contact, a represents the amount of overlap between the two particles in the normal direction, and b represents the tangential displacement of particle j.

**Figure 3:**
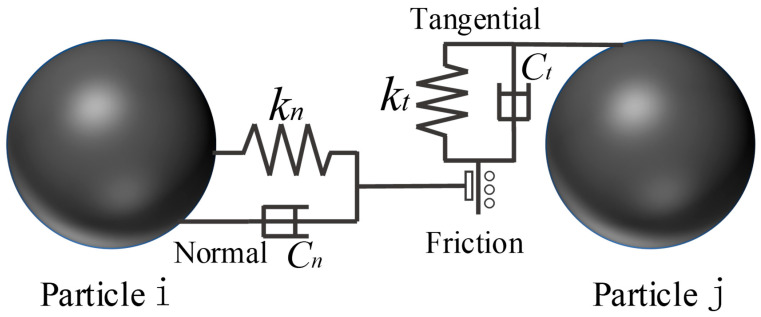
The contact mechanics of the soft-ball model.

Based on the soft-ball contact mechanics model, Figure 3 is a three-dimensional simplified model, which mainly introduces damping, spring, and friction coefficients between the two particles [17], and its normal force ($F_{nij}$) is shown in Equation (2).

(2) $$F_{nij}=-n[K_{n}a^{3/2}+C_{n}(V_{ni}-V_{nj})n]$$

*n*—the unit vector of particle *i* and particle *j*; *a*—the normal overlap of two particles; $a=r_{i}+r_{j}-g_{ij}$; $g_{ij}$—the distance between two particle centers; $V_{ni}$ and $V_{nj}$—the normal velocity of particle motion; $K_{n}$—the normal elastic coefficient; $C_{n}$—the normal damping coefficient. The unit vector (*n*) of particle *i* and particle *j*, the normal elastic coefficient ($K_{n}$), and the normal damping coefficient ($C_{n}$) are shown in Equations (3)–(5).

(3) $$n=\frac{r_{i}-r_{j}}{\left|r_{i}-\left.r_{j}\right|\right.}$$

(4) $$K_{n}=\frac{4}{3}E^{*}{\left(R^{*}\right)}^{1/2}$$

(5) $$C_{n}=2{\left(mK_{n}\right)}^{1/2}$$

*m*—the mass of the particle; E*—the effective modulus of elasticity; R*—the effective radius of the particle; E* and R* can be obtained from Equations (6) and (7).

(6) $$E^{*}=\frac{E_{i}E_{j}}{E_{i}(1-v_{i}^{2})+E_{j}(1-v_{j}^{2})}$$

(7) $$R^{*}=\frac{r_{i}r_{j}}{r_{i}+r_{j}}$$

where *E_i_* and *E_j_* are the elastic modulus of particle *i* and particle *j*, respectively, and *v_i_* and *v_j_* are the Poisson’s ratios of particle *i* and particle *j*, respectively.

The tangential force ($F_{tij}$) between particles is shown in Equation (8):

(8) $$F_{tij}=-(K_{t}b+C_{t}V_{t})$$

where *b* is the tangential displacement; $V_{t}$ is the particle contact point velocity; $K_{t}$ and $C_{t}$ are the tangential elastic coefficient and the damping coefficient, respectively. $K_{t}$ and $C_{t}$ can be obtained from Equations (9) and (10):

(9) $$K_{t}=8a^{1/2}G^{*}{\left(R^{*}\right)}^{1/2}$$

(10) $$C_{t}=2{\left(mK_{t}\right)}^{1/2}$$

where G* is the effective shear modulus, and its calculation formula is shown in Equation (11):

(11) $$G^{*}=\frac{G_{i}(2-v_{j})+G_{j}(2-v_{i})}{G_{i}G_{j}}$$

where *G_i_* and *G_j_* are the shear modulus of particle *i* and the particle *j*, respectively.

## 4. Random Generation of Three-Dimensional Powder Particles

**Figure 4:**
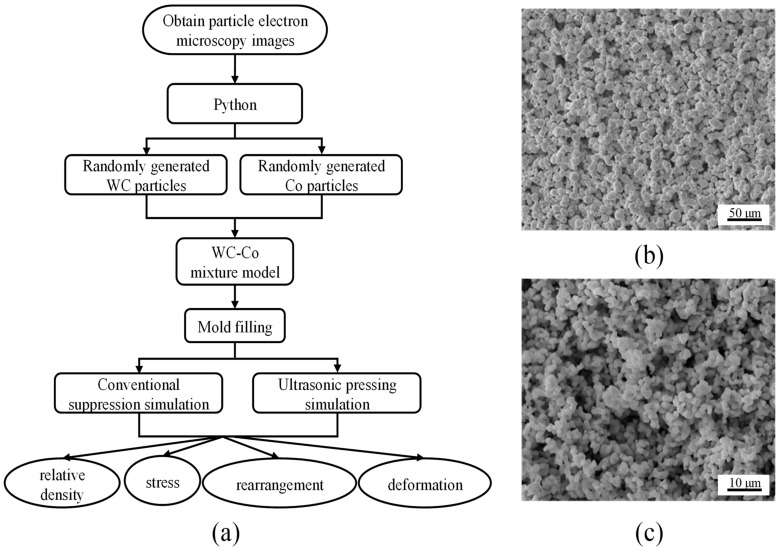
(**a**) The simulation flow chart. (**b**) The electron microscopic diagram of raw WC powder. (**c**) The electron microscopic image of raw Co powder.

In this paper, finite element simulation is carried out on the microscale particles to compare the mechanism of the particle rearrangement, deformation, and interaction under the assisted pressing process of ultrasonic vibration. The entire simulation process is shown in Figure 4a. First, the electron microscopic images of WC with a particle size of 5 μm and Co powder particles with a particle size of 1.2 μm are obtained, as shown in Figure 4b,c. It can be seen from the figures that WC and Co particles are similar to spherical particles. Therefore, a three-dimensional spherical model of two particle sizes is adopted to simulate the WC and Co particles.

**Figure 5:**
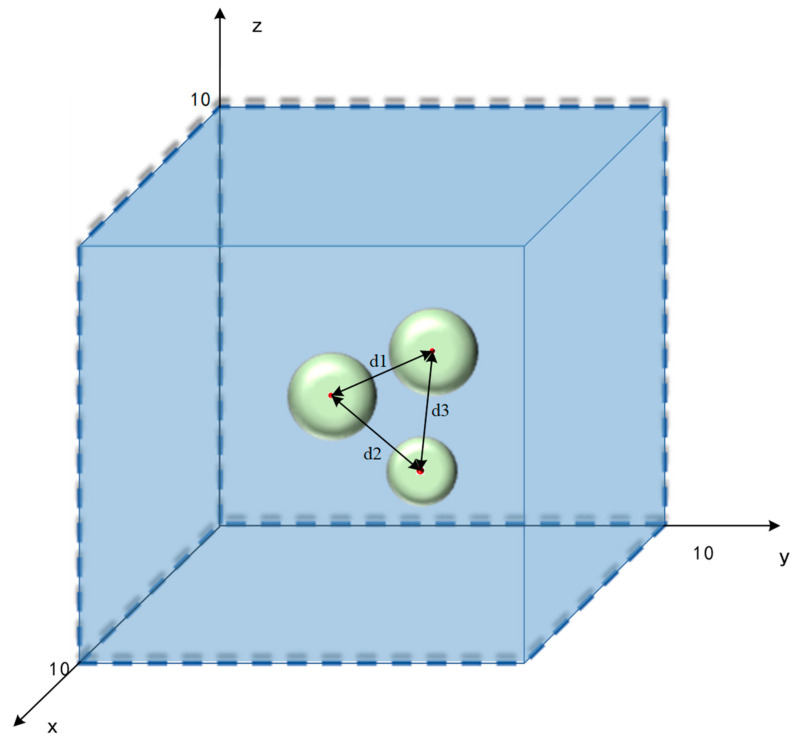
The schematic diagram of the particle drop.

The random generation of powder particles is performed through the ABAQUS and Python secondary development software. The main principles are as follows:

① To set the powder particle drop area, for example, randomly drop particles with radius R in a cube with length, width, and height of 10, as shown in Figure 5. Their spherical coordinates (*x*,*y*,*z*) must satisfy *R* ≤ *x* ≤ 10 − *R*, *R* ≤ *y* ≤ 10 − *R*, and *R* ≤ *z* ≤ 10 − *R*.
② To determine whether the randomly generated balls overlap:

Since two spherical particles of different particle sizes are generated, the radius of the two particle sizes is set to *R*_1_ and *R*_2_, respectively. The distance between the two spheres can be obtained by Formula (12):

(12) $$d=\sqrt{{\left(x_{i}-x_{j}\right)}^{2}+{\left(y_{i}-y_{j}\right)}^{2}+{\left(z_{i}-z_{j}\right)}^{2}}$$

The distance between any two particles should meet the following conditions, as shown in Equations (13) and (14):

(13) $$d_{1}\geq 2R_{1}$$

(14) $$d_{2}\geq R_{1}+R_{2}$$

③ The number and density of particles generated must be set and controlled.

## 5. Simulation Model and Parameter Setting

### 5.1. Material Attributes

The assignment of material properties is one of the essential steps of finite element simulation, and the ABAQUS software has a dedicated material library containing most of the commonly used materials, so the user can call or enter parameters directly. At the same time, the matching of parameter units is extremely important; the main reason is that the ABAQUS software has no fixed unit, so the user has to choose the corresponding matching unit for each quantity, and the unit of the final calculation result corresponds to the unit used. In this paper, the material properties of the corresponding parts are assigned as shown in Table 1.

### 5.2. Analysis Step Setting and Meshing

This work mainly studies the pressing process of WC-Co powder. The changes between powder particles are relatively complex, and there are multi-directional motions, rotations, collisions, contact deformations, etc. in the moving process, so this process is a highly nonlinear problem. In order to improve the efficiency of finite element calculation and save the simulation time, this paper adopts the dynamic-display analysis step to carry out the finite element simulation of the conventional pressing process and the ultrasonic-assisted pressing process.

**Figure 6:**
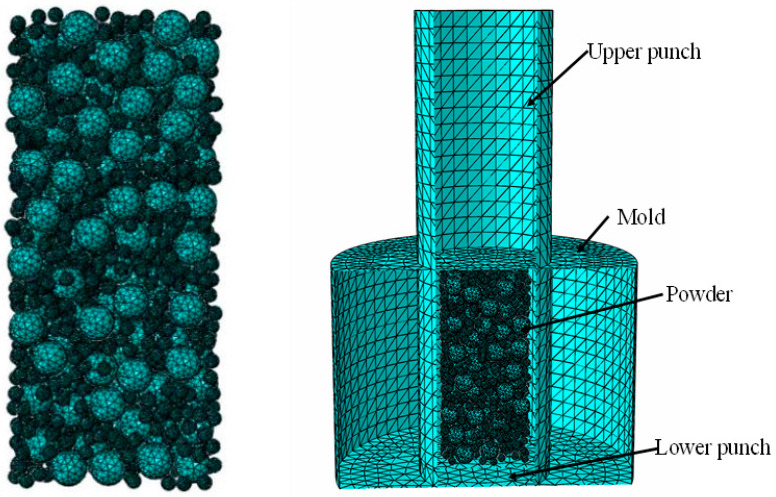
The WC-Co mixture and the press-model meshing.

In view of the complex particle movement in the powder pressing process, the mesh may be distorted, so the 4-node linear tetrahedral element mesh C3D4 in the dynamic-display analysis step is used in this paper. Since the parts have to be repeated many times when performing the material assignment and meshes in ABAQUS, the Python script is called to automate the material assignment and meshes, as shown in Figure 6.

### 5.3. Contact Properties and Boundary Conditions

When the contact properties are defined, the powder particles are deformable, while when the die and punch are set to rigid, compaction begins with the upper punch, and the die and lower punch are fixed. In the powder pressing process, the pressure is not all due to the deformation of the powder particles; part of it is consumed by the friction between the particles, the particles, and the mold wall, so that the pressure gradually decreases from top to bottom.

Here, under conventional pressing conditions, the friction coefficient of WC-Co is 0.2. Under ultrasonic pressing conditions, due to the anti-friction effect of ultrasound, the friction coefficient will be reduced by about 50%, according to Siegert’s research [18,19,20], so the friction coefficient is 0.1.

This work adopts axial unidirectional pressing and applies uniform downward displacement and sinusoidal vibration to the upper punch for a downward displacement of 24 μm and a pressing time 1 × 10^−2^ s, which causes displacement time curves to be coupled. At the same ultrasonic frequency of 20 kHz, the amplitudes of 1, 2, and 3 μm were applied, respectively. The effects of different amplitudes on the fluidity, filling density, and stress distribution of the WC-Co powder were investigated. To speed up the simulation, the mold and powder filling ranges were scaled, while other physical quantities were adjusted accordingly [21].

## 6. Results

### 6.1. Analysis of Particle Flow under the Conventional and the Ultrasonic Vibration-Assisted Compression Processes

**Figure 7:**
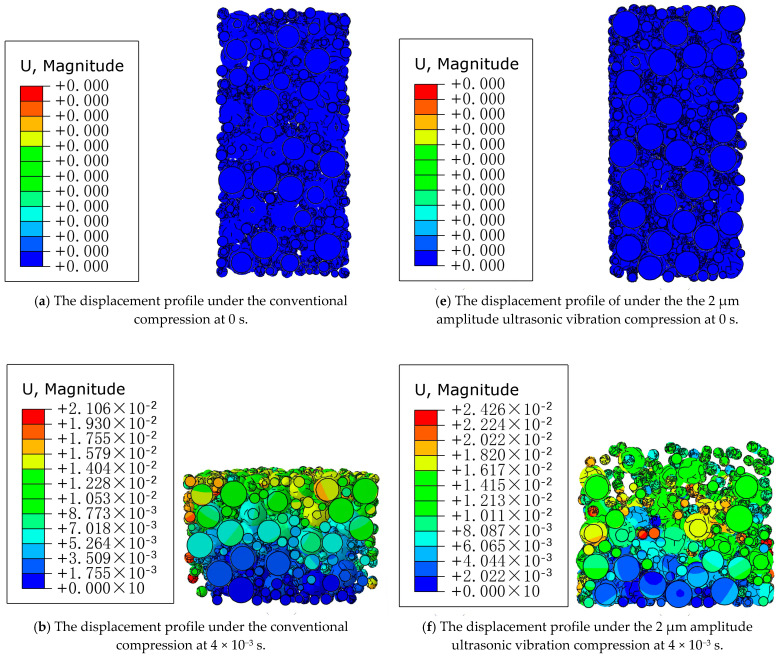

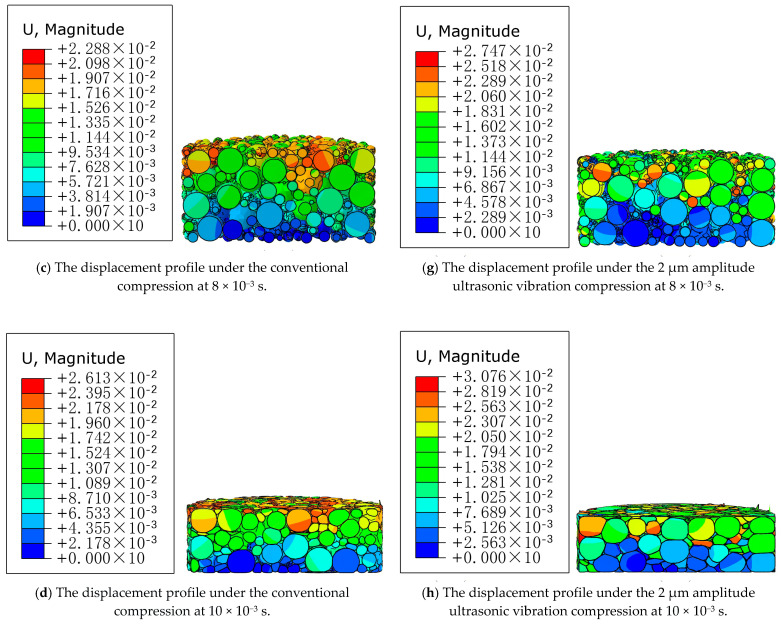
Displacement distribution cloud images at different times of the conventional pressing and ultrasonic pressing processes.

The fluidity characteristics of WC and Co particles are shown in Figure 7 under conventional and ultrasound-assisted compression conditions. In order to observe the particle fluidity more directly, a semi-sectional view is adopted. It can be seen from the figure that, without considering gravity, there are large pores between WC and Co particles in Figure 7a,e at the initial stage of compaction, and the contact force between particles is zero. When the upper punch moves down, the WC and Co particles near the upper punch first move to fill the pores, so contact slip occurs between the particles. Compared with conventional compaction, the particles move violently under the ultrasonic vibration, the particle fluidity is significantly enhanced, the arch bridge effect between particles is destroyed, and the fine particles quickly fill the pores between large particles in a short time to increase the density, and the uniformity of the particle size distribution is also improved [21,22]. As the upper punch continues to move down, the compact becomes more and more dense, and the particles gradually change from the original collision and sliding flow to mutual extrusion deformation. At this time, the increase in the compact density is mainly due to the plastic deformation of the particles. At the end of the pressing process, the height of the compact is significantly lower than that of the conventional pressing process under the action of ultrasonic vibration.

### 6.2. Effects of Ultrasonic Vibration Amplitudes on the Compact Density

**Figure 8:**
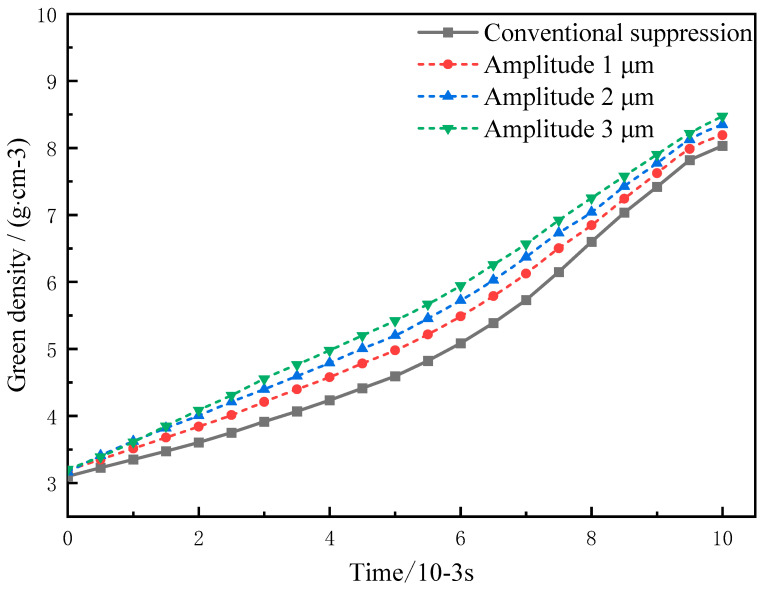
Effects of ultrasonic vibration amplitudes on the compact density.

The relationship curves between the ultrasonic amplitude and the compact density are obtained by comparison with the conventional pressing process under ultrasonic amplitudes of 1, 2, and 3 μm, respectively, as shown in Figure 8. As can be seen from Figure 8, since the initial density increase is related to the particle displacement, the compact density increases slowly. When the pressure increases to a certain extent, the compact density increases rapidly under the combined action of the plastic deformation and the displacement of the powder particles [23]. After applying different ultrasonic amplitudes, the displacement between particles is accelerated, and smaller particles fill the pores; thus, the compact density is significantly increased.

### 6.3. Effects of the Ultrasonic Vibration on the Stress Distribution of the Compact

**Figure 9:**
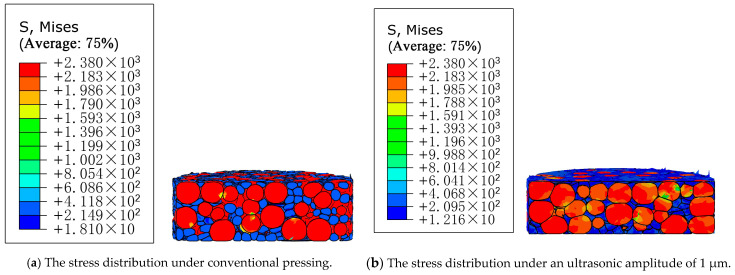

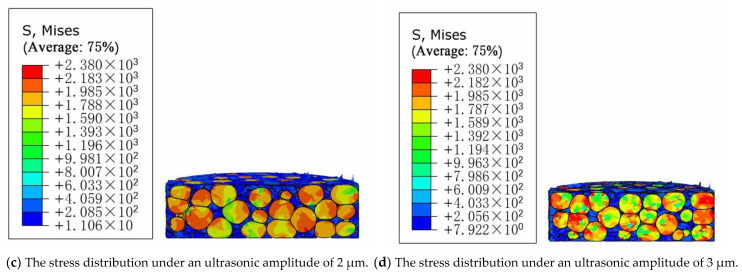
Mises stress distribution cloud images of WC and Co particles under conventional compression and different ultrasonic amplitudes.

The Mises stress distribution cloud of WC and Co particles is shown in Figure 9 under conventional compression and different ultrasonic amplitudes. It can be seen from the figure that the powder particles are deformed after pressing, because the contact between the particles will cause stress concentration, and the WC particles are harder, so the stress concentration is more obvious. However, after the ultrasonic vibration is applied, the stress at the contact between particles gradually decreases with the increase in ultrasonic amplitude, which indicates that ultrasonic vibration can change the stress distribution between particles and reduce the deformation stress between particles. Therefore, ultrasonic vibration can reduce the stress concentration between particles during the powder pressing process, thereby reducing the residual stress in the compact after the powder pressing, reducing the elastic after-effect [24], and improving the quality of the compact.

### 6.4. Experimental Verification

**Figure 10:**
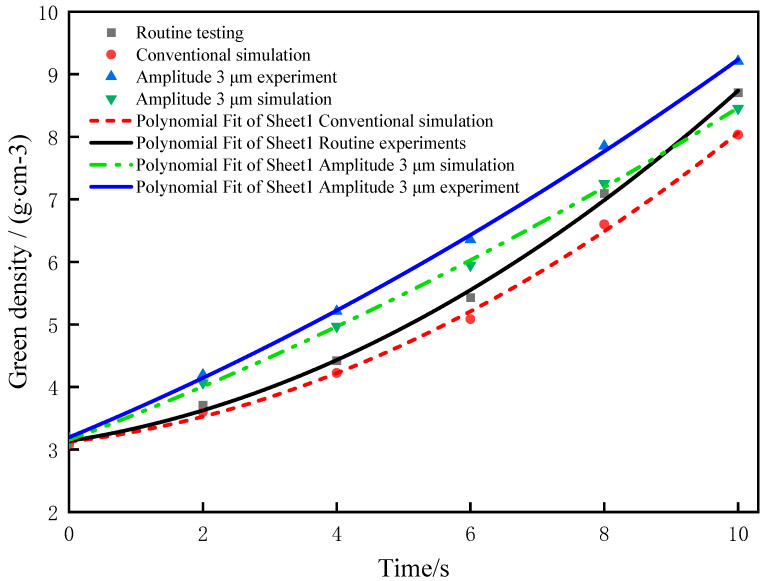
Results of the compression experiment and the simulation.

Experiments were carried out using two kinds of pressing processes to obtain the relationship between the pressing time and the compact density, and the experimental results were compared with the simulation results when the amplitude of vibration was 3 μm, as shown in Figure 10. It can be seen from the figure that the experimental and simulated compact density errors are not large, and the errors in the forming process are both lower than 10%, indicating that the finite element simulation and experimental results are relatively consistent [25]. When the density is greater than 5 g/cm^3^, the error between the experimental value and the simulation value is greater than 6%, mainly because the finite element simulation regards the friction coefficient between the particles and between the particles and the mold as a fixed value during the particle-forming process, while the friction coefficient between the particles and between the particles and the mold in the actual pressing process is a constantly changing process. The deformation and displacement of the particles in the middle and late pressing period are especially complicated, and it is difficult for a simulation to accurately reflect the forming process.

**Figure 11:**
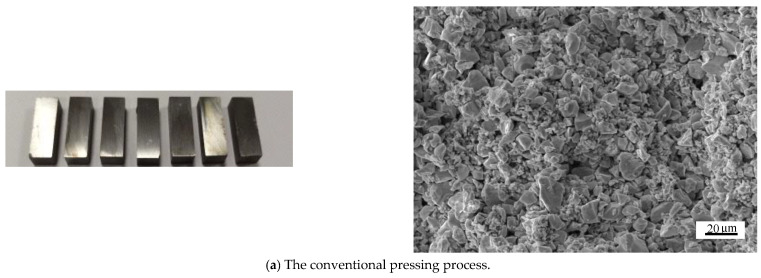

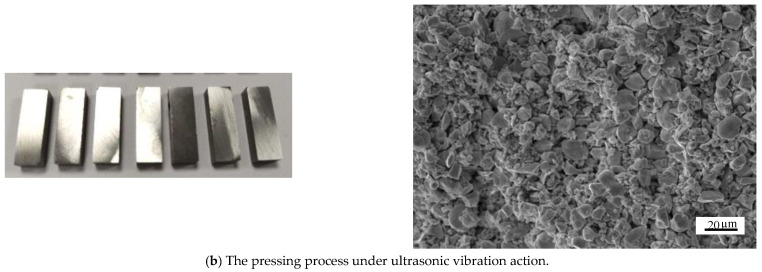
SEM of the longitudinal section of the compacts.

The cross-sectional microstructure of the compact under the two pressing conditions was observed and it was found that, as shown in Figure 11, the WC and Co particles were deformed correspondingly, and the particles changed from the original approximately circular state to a flat state, which is more consistent with the simulation effect. By comparing the cross sections of conventional and ultrasonic pressing compacts, it is found that the particle size distribution is more uniform under the ultrasonic pressing process, which is related to the acceleration of particle flow rearrangement under the ultrasonic action [26].

When the pressure is withdrawn, the powder particles will slowly return to their original state, and the compact will rebound and expand along the direction of the pressing force during and after demolding, and the compact size will increase. In order to characterize the size change after demolding, Chuxuan Chen [27] expressed as a percentage the increase in the size of the compact after demolding, as shown in Equation (15).

(15) $$\delta =\frac{\Delta H_{P}}{H_{P}}$$

*H_p_*—the pressed size; ∆*H_p_*—the increased size after demolding.

**Figure 12:**
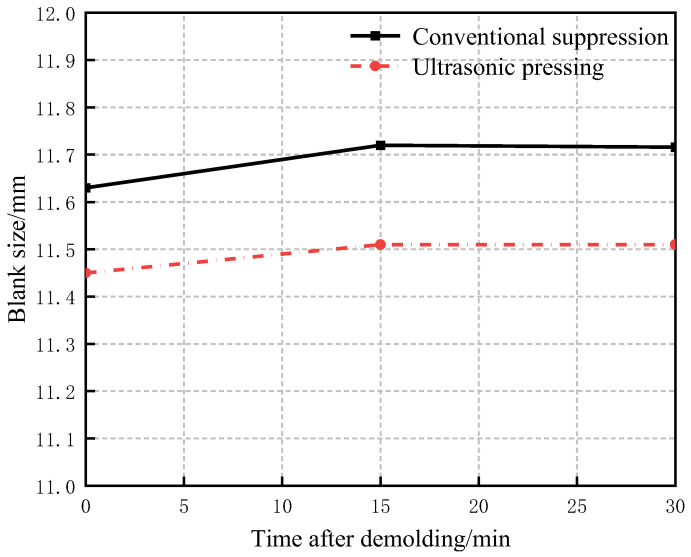
Changes in blank size after demolding.

**Figure 13:**
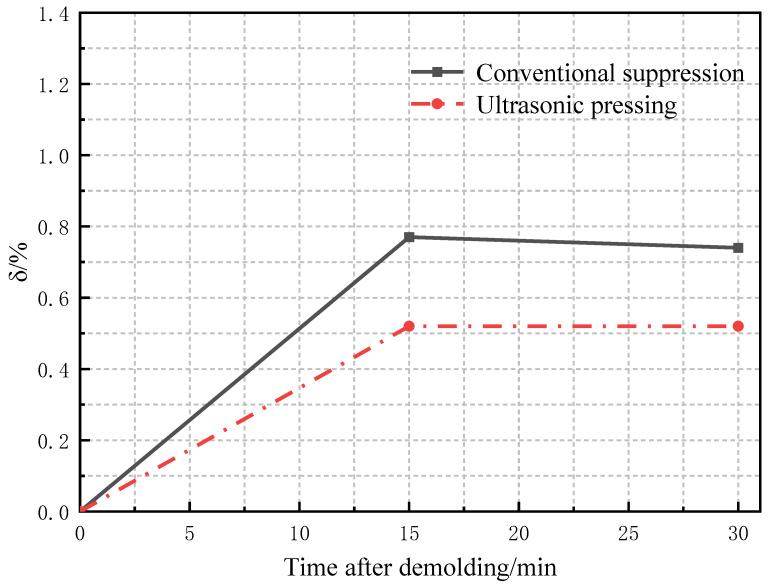
Changes of δ after demolding.

Under the same pressing force for the conventional and ultrasonic vibration pressing processes, the dimensions of the compact after demolding and the dimensions of the compact after standing for 15 min and 30 min were counted. Three different positions on the compact surface were measured for each dimension, and then the average value was taken to obtain the elastic after-effects of the compact after conventional pressing and ultrasonic-vibration-assisted pressing processes, as shown in Figure 12. The δ change, as shown in Figure 13, shows that the blank size increased rapidly for about 15 min after demolding. The results show that the elastic after-effect of the compact is reduced, and the dimensional instability is slightly improved, which is consistent with the simulation results in Figure 9, indicating that the residual internal stress is reduced due to the application of ultrasonic vibration during the pressing process [13].

## 7. Conclusions

In this paper, Python was used to randomly generate WC and Co particles, which were automatically utilized for material assignment and meshing to reduce the modeling time. Then, ABAQUS finite element simulation and experiments were conducted on the two pressing processes respectively, and the conclusions are as follows:

(1) The influence of ultrasonic vibration amplitude on the compact density is great, so to obtain the theoretical results, values of vibration amplitudes were set 1, 2, and 3 μm to verify that the compact density increases gradually with the increase in ultrasonic amplitude. Especially in the early stage of particle deformation, the fluidity between particles is relatively intense, and the particles quickly fill the pores, so the filling density of the powder is significantly increased compared with that under conventional pressing conditions.
(2) The use of ultrasonic vibration in powder pressing can effectively reduce the deformation stress between particles, reduce the residual stress in the compact after pressing, reduce the elastic after-effect, and improve the quality of the compact.
(3) The obtained experimental results verify the developed theoretical model of the pressing process. When the value of the ultrasonic vibration amplitude is 3 μm, the finite element simulation is consistent with the experimental results, but when the density is greater than 5 g/cm^3^, the error between the experimental value and the simulation value is greater than 6%, mainly because the finite element simulation regards the friction coefficient between particles and between particles and the mold as a constant value during the particle-forming process, while the friction coefficient between particles and between particles and the mold constantly changes during the actual pressing process.

## Figures and Tables

**Table 1:** The material properties of each component.

Performance Parameter	ABAQUS Unit	Mold	WC	Co
Density	Tonne/mm^3^	7.89 × 10^−9^	1.56 × 10^−9^	7.9 × 10^−10^
Modulus of elasticity	MPa	2.09 × 10^5^	7.14 × 10^5^	2.09 × 10^5^
Yield strength	MPa	no	2380	279
Poisson’s ratio	no	0.269	0.19	0.3

## Data Availability

The data presented in this study are available from the corresponding author upon reasonable request.
